# Physiological effects of a pre-anesthetic single dose of gabapentin on Xylazine/Ketamine anesthesia in rabbits

**DOI:** 10.1186/s12917-025-05142-8

**Published:** 2025-12-13

**Authors:** Mona Gadelrab, Mohamed El-Sherif, Mahmoud Atiya, Waleed Senosy, Mahmoud S. Salem, Mohamed Abdelkawi

**Affiliations:** 1 Department of Veterinary Surgery, Radiology, and Anesthesiology, Faculty of Veterinary Medicine, New Valley University, Al Kharga, New Valley, 27511 Egypt; 2 Deanship, Faculty of Veterinary Medicine, New Valley University, Al Kharga, New Valley, 27511 Egypt

**Keywords:** Gabapentin, Anesthesia, Rabbits, Ketamine, Xylazine

## Abstract

**Background:**

As common pets, rabbits are frequently brought to veterinarians for examination and medical care. Many diagnostic and surgical treatments require anesthesia, which is linked to a higher perioperative risk in rabbits than in dogs and cats.

**Objective:**

To assess the impact of a single 25 mg/kg oral dose of gabapentin on the depth and duration of anesthesia and hemodynamic stability of different anesthetic regimes.

**Methods:**

Twenty adult healthy New Zealand male rabbits weighing 2.5 ± 0.5 kg were used in this study randomly and equally divided using a computer-generated random number table into four groups: group A (gabapentin/xylazine/ketamine), group B (gabapentin/ketamine), group C (xylazine/ketamine), and group D (ketamine only). All experimental groups were evaluated through assessment of anesthetic depth, vital, hematological, and serum biochemical parameters.

**Results:**

Our data revealed the enhanced reflex suppression and prolonged recovery observed in the anesthetic regime of group (A) with short induction time and prolonged recovery with remarkable muscle relaxation.

**Limitations:**

The study was limited by a small sample size (*n* = 20), which may reduce the statistical power and generalizability of the findings. Additionally, postoperative pain assessment using validated scoring tools was not performed, and the results should be interpreted with caution regarding long-term analgesic efficacy.

**Conclusion:**

A single oral dose of gabapentin minimizes the ketamine’s catalepsy, reducing its induction time, delaying and improving the recovery.

## Background

Rabbits are widely kept as pets and frequently used in laboratory research [1, 2]. Normally, healthy rabbits are alert, active, and inquisitive about their surroundings, but their behavior changes when they are stressed or frightened. ​ This poses challenges for veterinarians, as unfamiliar environments, such as car rides or examination rooms, can provoke stress responses. ​ Additionally, handling, which is necessary during physical examinations and veterinary visits, can further contribute to their stress [3]. Stress in rabbits can lead to various negative outcomes [4]. stress contributes to higher anesthesia-related complications, with rabbits experiencing a greater mortality rate under anesthesia compared to cats and dogs [5]. ​ Medications that reduce anxiety are often used for small animals before events like vet appointments, car rides, or other potentially stressful situations, and they could also be advantageous for rabbits in similar circumstances [5].​.

Gabapentin, a gamma-aminobutyric acid (GABA) analog, has drawn interest in veterinary and human medicine due to its multimodal pharmacological effects, which include analgesic, anxiolytic, and anticonvulsant qualities [6]. While gabapentin was first created to treat epilepsy, it is now being utilized more and more as an adjuvant to treat neuropathic pain and perioperative anxiety in animals [6, 7]. In veterinary practice, gabapentin has been shown to enhance the effects of other anesthetic agents, reduce the required doses of induction drugs, and provide smoother recovery profiles [8, 9]. Despite its increasing use, the specific effects of gabapentin on anesthetic protocols in rabbits remain unknown, necessitating more research [10].

In rabbits, ketamine is frequently coupled with other drugs, such as xylazine or diazepam, to create balanced anesthesia [11]. However, ketamine alone can cause inadequate muscle relaxation and poor analgesia, necessitating the use of adjuncts to optimize its effects [12]. The combination of ketamine with gabapentin has shown promise in other species, but its efficacy and safety in rabbits have not been thoroughly studied.

This study aims to assess the impact of a single 25 mg/kg oral dose of gabapentin on the depth and duration of anesthesia, hemodynamic stability, and recovery quality of different anesthetic regimes. By investigating the potential benefits of gabapentin as an anesthetic adjunct, this study seeks to contribute to the development of safer and more effective anesthetic protocols for laboratory rabbits, ultimately improving animal welfare and the reliability of experimental outcomes.

## Methods

### Animal model and housing

Rabbits’ management adhered to the guidelines for the care and use of laboratory animals sanctioned by the Animal Care and Use Committee at the New Valley University, Faculty of Veterinary Medicine, New Valley, Egypt (registration number: VUSC-031-1-21). Twenty adult (five to six months age) apparently healthy New Zealand male rabbits, sourced from a private supplier (local market), weighting 2.5 ± 0.5 kg were used in this study which were allowed to acclimatize for three weeks before start of the experiment. All rabbits were apparently healthy without any history of musculoskeletal disorders. They were housed singly or in pairs in stainless steel cages with surface area 0.36 m² and were kept in humidity 55–60% with 12 h light/dark cycle and temperature 25 (± 3) °C. The rabbits were provided unrestricted access to a standard meal and water throughout the whole experiment. They received vaccination against Rabbit Hemorrhagic Disease virus (RHDV) with a 0.5 ml subcutaneous bivalent RHDV gel vaccine (SERVAC Co.; A.R.E) and were safeguarded against external and internal parasites using Dectomax (Zoetis Co.; A.R.E). The experimented rabbits were randomly and equally divided using a computer-generated random number table into four groups (*n* = 5); the sample size was pragmatically chosen based on feasibility and ethical considerations.

### Experimental design

According to the protocol of anesthetic regime, Rabbits were randomly assigned (www.randomizer.org) into four equals groups (5 rabbits each) labeled as A, B, C, and D.

Group A: in which the experimented rabbits were anesthetized by gabapentin (gaptin^®^, delta pharma company, Egypt) at dose 25 mg/kg body weight orally. After two hour a mixture of xylazine HCl (Xylaject: 2% sol. Adwia company, Egypt) at dose rate of 5 mg/kg and ketamine HCl (KETALITE^®^: ELICE Pharma; Pakistan) at dose rate 35 mg/kg were injected into Longissimus dorsi muscle.

Group B: in which the experimented rabbits were anesthetized by gabapentin (gaptin ^®^, delta pharma company, Egypt) at dose 25 mg/kg body weight orally. After two-hour animals were intramuscular injected by ketamine HCl (KETALITE^®^: ELICE Pharma; Pakistan) at dose rate 35 mg/kg.

Group C: in which the experimented rabbits were anesthetized by a mixture of xylazine HCl (Xylaject^®^: 2% sol. Adwia company, Egypt) at dose rate of 5 mg/kg and ketamine HCl (KETALITE^®^: ELICE Pharma; Pakistan) at dose rate 35 mg/kg were injected into Longissimus dorsi muscle.

Group D: in which the experimented rabbits were anesthetized by intramuscular injection of ketamine HCl (KETALITE^®^: ELICE Pharma; Pakistan) at dose rate 35 mg/kg.

### Methods of evaluation

Each regime was assessed through the following parameters:

1. Level of sedation, degree of responsiveness to noxious stimulus and depth of anesthesia
  1.1. Degree of responsiveness to noxious stimulus (analgesia)
  The response to a conventional noxious stimulus was used to evaluate the extent of responsiveness to noxious stimulus Hamad et al. [13],. A score of 0 indicated a normal response to a painful stimulus, while a score of 1 reflected mild analgesia characterized by a depressed reaction. A score of 2 denoted moderate analgesia, with no response to skin pricks, and a score of 3 represented complete analgesia, defined by the absence of response to muscle pricks.
  1.2 Level of sedation
  The degree of sedation was assessed by a trained observer who was blinded to group allocation, using a standardized scale ranging from 0 to 3 Valverde et al., [14]. A score of 0 indicated no sedation, while a score of 1 reflected mild sedation, characterized by reduced alertness but maintained activity. A score of 2 denoted moderate sedation, where the rabbit appeared drowsy and recumbent but retained the ability to walk. A score of 3 represented intense sedation, with the animal being very drowsy and unable to walk.
  1.3 Assessment of anesthetic depth
  The depth of anesthesia was assessed by evaluating several reflexes, including the palpebral reflex, pupillary light reflex, pedal reflex, anal reflex, swallowing reflex, and patellar reflex, in addition to observing muscle relaxation through jaw tone and eyeball rotation. Reflex responses were scored using the following system: (+++) indicated a normal reflex, (++-) represented a sluggish reflex, (+--) denoted a very sluggish reflex, and (---) signified an abolished reflex Smith, et al., [15]. Muscle relaxation was assessed qualitatively regarding the reflex suppression using a scale ranging from poor, fair, good, to very good.
  Level of sedation, degree of analgesia and depth of anesthesia scores were blindly recorded by two anesthesiologists
2. Determination of induction time and recovery time
  The induction time was recorded as the time from the start of anesthetic induction till developing the onset of anesthesia. while recovery times were determined by measuring the time for returning of voluntary swallowing, head lift, movement into sternal recumbency and voluntary unaided standing Hamad et al. [13],.
3. Assessment of vital parameters
  Vital parameters including heart rate (HR), respiratory rate (RR), and rectal temperature were evaluated. HR, and RR were measured using multi-parameter patient monitor (Yonker, China) Valverde et al. [14],.
4. Assessment of hematological and serum biochemical parameters: Bassan et al., [16]
  A Blood was withdrawn via middle ear vein using a 23-gauge needle and a five ml plastic syringe. A 0.5 ml of blood sample from were obtained for evaluation of hematological parameters in this study included red blood cell count (RBCs), packed cell volume (PCV), hemoglobin concentration (Hb), total leucocytic count (TLC), and platelet count.
  A 0.5 ml of blood sample from middle ear vein were assessed for concentrations of urea, creatinine, and serum enzymatic activities of alanine transaminase (ALT), aspartate transaminase (AST) and Alkaline phosphatase (ALP). All these variables were determined by spectrophotometer and commercial kits supplied by Biodiagnostic (Egypt).
  Sampling was staggered to minimize physiological impact, and micro-sampling techniques were employed. To mitigate iatrogenic effects, rabbits received subcutaneous fluids at a dose of 5 ml/kg post-procedure. Baseline measurements were standardized across all groups and defined as time zero, prior to any drug administration.

### Statistical analyses

Continuous outcomes (vital signs, hematology, biochemistry, induction and recovery times) were summarized as mean ± SD and analyzed using two-way repeated-measures ANOVA with fixed effects for Group (A–D), Time, and the Group×Time interaction. Normality of residuals was assessed with Shapiro–Wilk and Q–Q plots; homoscedasticity by residual vs. fitted plots; sphericity by Mauchly’s test; when sphericity was violated, Greenhouse–Geisser correction was applied. Post-hoc pairwise comparisons used Tukey adjustment.

Ordinal endpoints (analgesia score, sedation score, reflex suppression score, muscle-relaxation grade) were summarized as median [IQR] and analyzed using a linear mixed-effects model on rank-transformed scores with Group, Time, and Group×Time as fixed effects and a random intercept for Rabbit ID (fit in R/lme4). As a sensitivity analysis, we fit an ordinal logistic mixed-effects model; inference was concordant, so we report the rank-LMM results. Post-hoc contrasts on model-based estimated marginal means were adjusted for multiplicity (Tukey). Statistical significance was set at *p* ≤ 0.05 (two-sided).

## Results

### Analgesic effects

At 15 min, Group A reached the maximum analgesia (median [IQR] = 3 [3–3]) and sustained scores ≥ 2 through 75 min. Group C showed high early analgesia (15 min: 3 [2–3]), whereas Group B peaked at 30 min (2 [2–3]) and then declined to 1 by 75–90 min. Group D never exceeded a median score of 2 and fell to 0 by 90 min (Table 1).

**Table 1:** Analgesic scores over time among the tested groups

Time	Group A	Group B	Group C	Group D	*p*-value
Baseline	0	0	0	0	-
Start time	1 (1–2)	1 (1–2)	0	0	0.029*
15 min	3 (3–3)	2 (2–2)	3 (2–3)	2 (2–2)	0.001*
30 min	3 (3–3)	3 (2–3)	2 (2–3)	2 (2–2)	0.007*
45 min	3 (2–3)	2 (2–2)	2 (2–2)	1 (1–2)	< 0.001*
60 min	3 (2–3)	2 (1–2)	2 (1–2)	1 (0–1)	< 0.001*
75 min	2 (2–2)	1 (1–1)	1 (1–2)	0 (0–1)	< 0.001*
90 min	2 (1–2)	1 (0–1)	1 (1–1)	0	0.002*

The rank‑based linear mixed‑effects model (fixed effects: Group, Time, Group×Time; random intercept: Rabbit ID) showed significant Group, Time, and Group×Time effects (all *p* < 0.001). Model‑based post‑hoc contrasts (Tukey‑adjusted) indicated A > C > B > D at 15–60 min (all adjusted *p* < 0.05). Effect size placeholder: Δ(EMM) A–D at 30 min = ⟨value⟩ (95% CI ⟨lower⟩–⟨upper⟩).

## Sedative effects

At 15 min, Groups A and C showed intense sedation (both 3 [3–3]), Group B was moderate (2 [2–3]), and Group D rarely exceeded 2 (2 [1–2]). Group A maintained 3 [3–3] through 45–60 min and remained ≥ 2 at 75–90 min; Group C declined after 60 min (2 [2–2] at 60 min; 1 [1–2] at 90 min). Group B dropped to 1 [1–1] by 60 min, and Group D to 0 [0–1] by 75–90 min (Table 2).

**Table 2:** Sedative scores over time among the tested groups

Time	Group A	Group B	Group C	Group D	*p*-value
Baseline	0	0	0	0	-
Start time	1 (0–1)	1 (0–1)	0	0	0.029
15 min	3 (3–3)	2 (2–3)	3 (3–3)	2 (1–2)	0.001
30 min	3 (3–3)	2 (2–3)	3 (3–3)	2 (2–2)	0.001
45 min	3 (3–3)	2 (1–2)	3 (2–3)	1 (1–2)	0.001
60 min	3 (3–3)	1 (1–1)	2 (2–2)	1 (0–1)	0.001
75 min	2 (2–3)	1 (0–1)	2 (2–2)	0	0.003
90 min	2 (2–3)	0 (0–1)	1 (1–2)	0	0.002

Mixed-model analysis showed significant Group, Time, and interaction effects (*p* < 0.001). Post-hoc contrasts: A > B and A > D at all time points (p_adj < 0.001); A > C after 60 min (p_adj < 0.01). Time to 50% reduction: A = 82 min (95% CI ⟨lower⟩–⟨upper⟩) vs. C = 48 min (95% CI ⟨lower⟩–⟨upper⟩), log-rank *p* = 0.003.

## Anesthetic depth (reflex suppression)

Reflexes were abolished in Groups A, B, and C at 30–45 min, with Group A showing abolished responses from 15 to 45 min, recovering to very sluggish by 60–75 min, and to sluggish by 90 min. Group D showed the least suppression, maintaining stronger reflexes through observation (Table 3).

**Table 3:** Testing the reflexes response of deferent tested groups throughout the observation period

Time	Base line	Start time	15 min	30 min	45 min	60 min	75 min	90 min
Group (A)	+++	+ + -	- - -	- - -	- - -	+ - -	+ - -	+ + -
Group (B)	+++	+ + -	+ - -	- - -	+ - -	+ - -	+ + -	+ + -
Group (C)	+++	+++	++-	- - -	- - -	+ - -	+ + -	+ + -
Group (D)	+++	+++	+ + -	+ - -	+ + -	+ + -	+++	+ + +
p-value	-	0.029	0.001	0.002	0.02	0.36	0.36	0.029

(+++)=3: Reflex normal, (+ + -)=2: Reflex sluggish, (+ - -)=1: Reflex very sluggish, (- - -)=0: Reflex abolished

The rank‑based mixed model confirmed significant Group, Time, and interaction effects (*p* < 0.01 at reflex‑specific peaks); Tukey‑adjusted contrasts favored A over B and C during 15–45 min (p adj < 0.05).

## Muscle relaxation

Muscle relaxation peaked at “very good” in Groups A and C at 30–45 min and remained “good” at 60 min; Group B peaked earlier (30 min) and declined to “fair/poor” by 75–90 min; Group D reached “good” at 30–45 min then regressed (Table 4).

**Table 4:** Degree of muscle relaxation of deferent tested groups throughout the observation period

Time	Base line	Start time	15 min	30 min	45 min	60 min	75 min	90 min
Group (A)	poor	fair	Very good	Very good	Very good	good	good	fair
Group (B)	Poor	fair	good	Very good	good	good	fair	poor
Group (C)	poor	poor	good	Very good	Very good	good	fair	fair
Group (D)	poor	poor	fair	good	fair	fair	poor	poor
p-value	-	0.029*	0.001*	0.002*	0.002*	0.36	0.36	0.029*

Scores: Poor=0, Fair=1, Good=2, Very Good=3. (*) indicates significant difference p<0.05

Rank‑based mixed‑model inference supported significant Group and Time effects (*p* < 0.01), with adjusted post‑hoc contrasts favoring A and C over B and D during 15–60 min (p adj < 0.05).

## Induction and recovery times

Induction time was shortest in Group A (5.33 ± 1.50 min; 95% CI 3.47–7.19) and longest in Group D (14.60 ± 3.50 min; 95% CI 10.25–18.95). Group B required 10.00 ± 2.60 min (95% CI 6.77–13.23), and Group C 11.33 ± 2.00 min (95% CI 8.85–13.81). Recovery was longest in Group A (142.66 ± 11.23 min; 95% CI 128.72–156.60) and shortest in Group D (22.30 ± 2.50 min; 95% CI 19.20–25.40), with Group B (55.30 ± 5.60 min; 95% CI 48.35–62.25) and Group C (82.30 ± 6.20 min; 95% CI 74.60–90.00) in‑between (Table 5).

**Table 5:** Determination of induction time and quality of induction, recovery time and quality

Groups	Induction time (min)	Recovery time (min)
Group A	5.33 ± 1.5	142.66 ± 11.23
Group B	10 ± 2.6	55.3 ± 5.6
Group C	11.33 ± 2	82.3 ± 6.2
Group D	14.6 ± 3.5	22.3 ± 2.5
p-value	< 0.001*	< 0.001*

Two‑way RM‑ANOVA showed significant Group effects for both outcomes (*p* < 0.001) with Tukey post‑hoc differences consistent with the above ordering. *Effect size placeholder*: partial η² = ⟨value⟩.

The induction time was recorded as the time from the start of anesthetic induction till developing the onset of anesthesia (loss of palpebral reflex, loss of jaw tone and lack of tongue withdrawal).

The recovery was assessed by the existence of the righting reflex (ability to return sternal position by own).

## Vital parameters

**Fig. 1:**
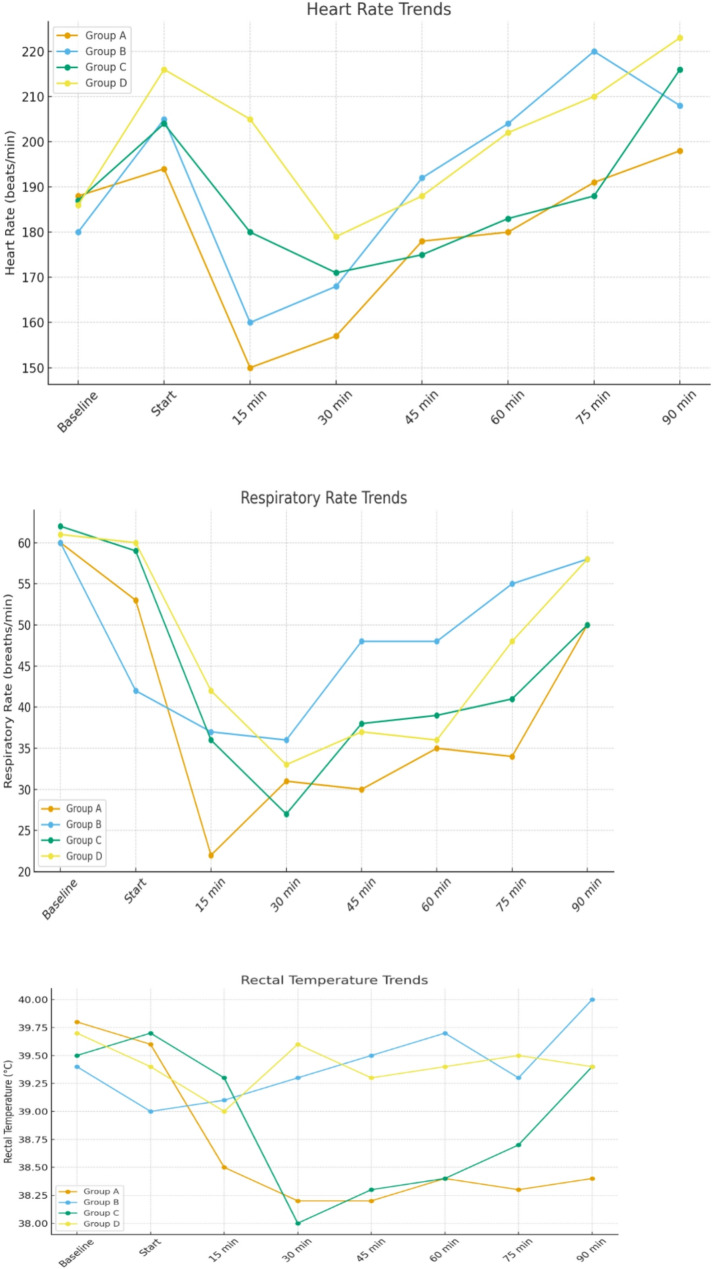
Changes of the heart rate, respiratory rate and rectal temperature among tested group over the time

At 15 min (near peak anesthetic depth). Heart rate (bpm): Group A 150 ± 5.5 (95% CI 143.17–156.83) vs. Group D 205 ± 9.6 (95% CI 193.08–216.92). Groups B and C were intermediate. Respiratory rate (breaths/min): Group A 22 ± 4.8 (95% CI 16.04–27.96) vs. Group D 42 ± 8.2 (95% CI 31.82–52.18). Rectal temperature (°C): Group A 38.5 ± 0.3 (95% CI 38.13–38.87) vs. Group D 39.0 ± 0.9 (95% CI 37.88–40.12). (Table 6; Fig. 1).

**Table 6:** Showing the vital parameter of all tested groups throughout the observation period

Vital Sign	Group	Baseline	Start time	15 min	30 min	45 min	60 min	75 min	90 min
**Heart Rate/min**	**A**	188 ± 9.9	194 ± 7.1	150 ± 5.5	157 ± 8.4	178 ± 11.2	180 ± 9.6	191 ± 10.3	198 ± 8.8
	**B**	180 ± 6.7	205 ± 10.1	160 ± 9.6	168 ± 8.4	192 ± 6.9	204 ± 11.2	220 ± 9.1	208 ± 10.3
	**C**	187 ± 8.7	204 ± 9.2	180 ± 12.5	171 ± 10.1	175 ± 11.7	183 ± 8.9	188 ± 13.3	216 ± 11.8
	**D**	186 ± 5.7	216 ± 10.3	205 ± 9.6	179 ± 11.2	188 ± 8.3	202 ± 10.9	210 ± 12.6	223 ± 9.5
	**p-value**	**0.365**	0.012*	< 0.001*	0.040*	0.027*	0.015*	0.004*	0.003*
**Respiratory Rate/min**	**A**	60 ± 3.2	53 ± 5.9	22 ± 4.8	31 ± 2.8	30 ± 1.6	35 ± 3.5	34 ± 2.2	50 ± 4.9
	**B**	60 ± 5.4	42 ± 3.3	37 ± 2.6	36 ± 2.2	48 ± 4.1	48 ± 3.8	55 ± 2.7	58 ± 2.9
	**C**	62 ± 1.2	59 ± 5.4	36 ± 6.1	27 ± 8.7	38 ± 4.8	39 ± 7.9	41 ± 4.6	50 ± 7.1
	**D**	61 ± 3.1	60 ± 4.5	42 ± 8.2	33 ± 6.9	37 ± 7.3	36 ± 9.1	48 ± 5.8	58 ± 4.2
	**p-value**	**0.548**	< 0.001*	< 0.001*	**0.070**	0.001*	0.013*	< 0.001*	0.004*
**Rectal temperature (°C)**	**A**	39.8 ± 0.3	39.6 ± 0.5	38.5 ± 0.3	38.2 ± 0.14	38.2 ± 0.4	38.4 ± 0.3	38.3 ± 0.2	38.4 ± 0.3
	**B**	39.4 ± 0.2	39.0 ± 0.3	39.1 ± 0.3	39.3 ± 0.4	39.5 ± 0.2	39.7 ± 0.6	39.3 ± 0.3	40.0 ± 0.7
	**C**	39.5 ± 0.3	39.7 ± 0.9	39.3 ± 0.3	38.0 ± 0.7	38.3 ± 0.4	38.4 ± 0.6	38.7 ± 0.2	39.4 ± 0.3
	**D**	39.7 ± 0.5	39.4 ± 0.4	39.0 ± 0.9	39.6 ± 0.8	39.3 ± 0.7	39.4 ± 0.3	39.5 ± 0.6	39.4 ± 0.8
	**p-value**	**0.090**	**0.178**	0.005*	< 0.001*	0.001*	0.013*	0.009*	0.042*

Two‑way RM‑ANOVA confirmed significant between‑group differences at multiple time points for HR and RR (15–30 min: *p* < 0.001), and for temperature when hypothermia occurred in xylazine‑containing regimens (*p* ≤ 0.01), with Greenhouse–Geisser correction applied when sphericity was violated.

## Hematological and serum biochemical changes

### Hematological changes

Across time, hematological indices largely remained within normal ranges in all groups (Table 7). Hemoglobin (g/dL): Group A 11.1 ± 0.2 at 15 min; Group C 10.4 ± 2.1 at 15 min. RBCs (×10⁶/µL): Group A 4.8 ± 0.5 at 15 min; Group C 5.1 ± 0.89 at 15 min.

**Table 7:** Showing the hematological parameter of all tested groups throughout the observation period

Parameter	Group	Baseline	Start time	15 min	30 min	45 min	60 min	75 min	90 min	Normal Range
**Hgb (g/dL)**	**A**	12.2 ± 0.2	11.2 ± 0.3	11.1 ± 0.2	11.9 ± 0.4	11.3 ± 0.2	11.0 ± 0.2	11.2 ± 0.1	11.6 ± 0.3	**8–15**
	**B**	10.5 ± 0.6	11.1 ± 0.9	11.9 ± 0.25	10.8 ± 1.1	11.8 ± 0.4	10.7 ± 0.8	10.1 ± 0.7	10.0 ± 0.3	
	**C**	11.2 ± 0.5	12.0 ± 1.2	10.4 ± 2.1	11.9 ± 1.8	11.2 ± 1.5	9.3 ± 1.3	10.4 ± 2.1	11.8 ± 1.4	
	**D**	11.8 ± 0.4	12.0 ± 0.9	11.4 ± 0.8	11.2 ± 1.1	11.8 ± 1.3	11.5 ± 0.7	11.2 ± 1.0	11.3 ± 0.9	
	**p-value**	0.001*	**0.135**	**0.260**	**0.485**	**0.456**	0.005*	**0.105**	0.002*	
**RBCs (×10⁶/mcL)**	**A**	6.1 ± 0.3	5.4 ± 0.23	4.8 ± 0.5	5.8 ± 0.3	5.5 ± 0.6	5.4 ± 0.3	5.7 ± 0.3	5.7 ± 0.2	**4–7**
	**B**	5.2 ± 0.3	5.4 ± 0.9	5.7 ± 0.7	5.4 ± 0.2	5.6 ± 0.4	5.5 ± 0.8	4.1 ± 1.1	4.9 ± 0.6	
	**C**	5.5 ± 0.4	5.9 ± 0.67	5.1 ± 0.89	5.6 ± 0.58	5.6 ± 0.72	4.6 ± 0.44	5.1 ± 0.63	5.5 ± 0.68	
	**D**	5.7 ± 0.3	5.7 ± 0.3	5.3 ± 0.2	5.3 ± 0.3	5.4 ± 0.5	5.6 ± 0.2	5.1 ± 0.4	5.7 ± 0.6	
	**p-value**	0.002*	**0.302**	**0.054**	**0.216**	**0.766**	0.018*	0.008*	0.041*	
**MCV (fL)**	**A**	67.2 ± 6.6	68.5 ± 9.1	72.9 ± 11.2	65.5 ± 5.4	67.6 ± 8.3	64.8 ± 4.7	66.1 ± 5.9	64.9 ± 4.6	**58–67**
	**B**	63.7 ± 7.2	68.5 ± 11.9	70.2 ± 5.9	64.8 ± 10.3	69.6 ± 8.5	72.1 ± 12.1	78.0 ± 6.7	65.3 ± 9.4	
	**C**	63.2 ± 4.1	66.1 ± 3.2	66.7 ± 2.5	69.6 ± 1.6	66.1 ± 3.7	65.2 ± 1.9	66.7 ± 2.4	67.3 ± 3.1	
	**D**	66.5 ± 2.3	58.4 ± 5.1	60.0 ± 4.7	61.6 ± 4.9	62.2 ± 5.6	60.9 ± 3.9	61.6 ± 4.3	63.0 ± 3.7	
	**p-value**	**0.363**	0.019*	0.012*	**0.146**	**0.171**	**0.096**	0.001*	**0.225**	
**MCH (pg)**	**A**	20.0 ± 2.4	20.6 ± 1.7	25.1 ± 1.9	20.5 ± 2.1	21.4 ± 1.7	20.4 ± 1.6	20.3 ± 2.2	20.4 ± 1.1	**17.1–23.5**
	**B**	21.9 ± 3.2	20.6 ± 4.9	20.9 ± 2.9	20.0 ± 5.3	21.4 ± 3.5	22.3 ± 3.2	24.6 ± 4.4	20.4 ± 3.8	
	**C**	20.2 ± 0.7	20.3 ± 1.5	20.4 ± 1.9	21.3 ± 2.1	20.0 ± 1.7	20.2 ± 1.6	20.4 ± 1.4	21.5 ± 1.8	
	**D**	20.6 ± 0.9	21.1 ± 1.2	21.3 ± 1.1	21.5 ± 0.9	21.9 ± 1.4	21.6 ± 1.1	22.0 ± 0.8	22.2 ± 1.2	
	**p-value**	**0.383**	**0.962**	0.008*	**0.831**	**0.466**	**0.254**	**0.071**	**0.368**	
**MCHC (g/dL)**	**A**	29.8 ± 3.1	30.0 ± 2.8	31.9 ± 2.3	31.3 ± 1.9	30.6 ± 2.4	31.4 ± 1.6	30.8 ± 1.5	31.4 ± 2.9	**29–37**
	**B**	33.0 ± 4.1	30.0 ± 5.5	29.8 ± 3.7	30.9 ± 2.9	30.8 ± 4.3	30.2 ± 3.2	31.6 ± 5.0	31.2 ± 4.8	
	**C**	30.5 ± 0.7	30.8 ± 2.3	30.6 ± 2.1	30.3 ± 1.7	30.3 ± 1.5	31.0 ± 1.9	30.6 ± 2.0	31.9 ± 2.2	
	**D**	31.1 ± 0.4	30.8 ± 0.1	31.5 ± 0.2	31.0 ± 0.1	30.3 ± 0.6	30.8 ± 0.4	31.1 ± 0.3	32.7 ± 0.5	
	**p-value**	**0.075**	**0.948**	**0.614**	**0.833**	**0.968**	**0.834**	**0.955**	**0.805**	
**Plt (×10³/mcL)**	**A**	356 ± 56	341 ± 42	299 ± 57	352 ± 69	323 ± 34	382 ± 40	301 ± 52	279 ± 61	**250–650**
	**B**	337 ± 42	341 ± 55	295 ± 38	351 ± 57	246 ± 49	294 ± 61	297 ± 52	263 ± 35	
	**C**	433 ± 43	404 ± 32	368 ± 51	391 ± 47	544 ± 63	409 ± 55	498 ± 43	461 ± 35	
	**D**	407 ± 37	432 ± 22	401 ± 34	426 ± 27	395 ± 48	483 ± 38	463 ± 23	425 ± 29	
	**p-value**	0.001*	< 0.001*	0.001*	0.006*	< 0.001*	< 0.001*	< 0.001*	< 0.001*	
**WBCs (×10³/mcL)**	**A**	8.9 ± 0.7	8.3 ± 0.3	6.1 ± 0.6	10.3 ± 1.1	8.8 ± 0.7	6.8 ± 0.3	9.1 ± 0.9	9.8 ± 0.5	**6–12**
	**B**	7.8 ± 1.2	8.3 ± 0.8	7.9 ± 0.6	10.6 ± 1.4	8.1 ± 0.9	8.8 ± 0.7	9.4 ± 1.1	8.1 ± 1.3	
	**C**	7.7 ± 0.9	7.9 ± 1.3	7.1 ± 0.9	6.9 ± 1.4	8.1 ± 1.9	6.9 ± 0.7	7.9 ± 0.8	7.2 ± 1.1	
	**D**	7.3 ± 0.7	7.5 ± 0.4	7.9 ± 0.4	8.4 ± 0.8	8.9 ± 0.6	8.2 ± 0.7	7.8 ± 0.3	8.1 ± 0.5	
	**p-value**	0.042*	**0.279**	0.003*	0.001*	**0.310**	0.002*	**0.054**	0.012*	

Two way RM ANOVA identified sporadic time and group effects (Hgb at 60–90 min, RBCs at 60–90 min; *p* ≤ 0.05), but values remained within species typical ranges.

### Biochemical changes

Values were generally within reference limits, with transient, protocol‑related peaks (Table 8). AST (IU/L): Group C peaked at 75 min (83 ± 13; 95% CI 66.86–99.14); Group D showed an earlier, moderate rise at 30 min (70 ± 8.7). ALT (IU/L): Group A showed a moderate peak at 45 min (70 ± 13; 95% CI 53.86–86.14); Groups B and C remained lower at corresponding times. Urea (mg/dL): Group D at 75 min 35 ± 2.1 (95% CI 32.39–37.61); others were within 20–38 across time. Creatinine (mg/dL): Group A at 60 min 0.76 ± 0.27 (95% CI 0.43–1.10); similar low‑normal values appeared in other groups across time.

**Table 8:** Biochemical parameters of all groups at different evaluation times

Parameter	Group	Baseline	Start time	15 min	30 min	45 min	60 min	75 min	90 min	Normal Range
**AST (IU/L)**	**A**	49 ± 11	55 ± 13	53 ± 9	49 ± 15	67 ± 6	51 ± 8	53 ± 10	34 ± 7	**35–130**
	**B**	39 ± 12	38 ± 16	36 ± 8	42 ± 14	56 ± 11	68 ± 9	78 ± 17	48 ± 13	
	**C**	45 ± 11	51 ± 11	47 ± 10	46 ± 8	64 ± 9	50 ± 7	83 ± 13	46 ± 12	
	**D**	39 ± 19	66 ± 4.9	61 ± 11.4	70 ± 8.7	62 ± 7.2	65 ± 5.6	47 ± 9.1	56 ± 6.3	
	**p-value**	**0.62**	**0.006** ^*^	**0.018** ^*^	**0.012** ^*^	**0.01** ^*^	**0.055**	**0.001** ^*^	**0.17**	
**ALT (IU/L)**	**A**	59 ± 6	54 ± 13	58 ± 15	50 ± 17	70 ± 13	65 ± 11	61 ± 9	58 ± 7	**45–80**
	**B**	52 ± 15	49 ± 10	53 ± 14	25 ± 7	67 ± 18	55 ± 16	45 ± 9	45 ± 13	
	**C**	55 ± 4	38 ± 5.2	33 ± 6.8	47 ± 4.7	43 ± 5.9	39 ± 6.3	48 ± 7.2	35 ± 6.6	
	**D**	57 ± 8	59 ± 12.4	68 ± 9.2	55 ± 11.5	60 ± 13.1	58 ± 7.3	53 ± 8.9	55 ± 7.7	
	**p-value**	**0.51**	**0.06** ^*^	**0.001** ^*^	**0.001** ^*^	**0.007** ^*^	**0.048** ^*^	**0.059**	**0.02** ^*^	
**ALP (IU/L)**	**A**	25 ± 15	29 ± 7	28 ± 12	19 ± 11	29 ± 5	31 ± 9	20 ± 11	18 ± 10	**12–96**
	**B**	25 ± 7	22 ± 9	33 ± 18	37 ± 16	48 ± 11	35 ± 12	33 ± 14	41 ± 8	
	**C**	26 ± 1	17 ± 2.4	23 ± 2.9	21 ± 3.6	19 ± 2.7	25 ± 3.4	20 ± 3.1	18 ± 2.2	
	**D**	24 ± 5	28 ± 6.1	24 ± 5.2	27 ± 6.9	22 ± 4.5	31 ± 4.3	23 ± 6.2	26 ± 5.7	
	**p-value**	**0.87**	**0.055**	**0.2**	**0.1**	**0.004** ^*^	**0.171**	**0.23** ^*^	**0.006** ^*^	
**CREAT (mg/dL)**	**A**	0.89 ± 0.13	0.99 ± 0.25	0.96 ± 0.21	0.9 ± 0.18	1.1 ± 0.29	0.76 ± 0.27	0.85 ± 0.12	0.93 ± 0.22	**0.5–2.5**
	**B**	0.65 ± 0.12	0.74 ± 0.15	0.86 ± 0.11	0.8 ± 2.9	1.2 ± 0.21	0.79 ± 0.27	0.9 ± 0.13	0.79 ± 0.19	
	**C**	0.68 ± 0.16	0.61 ± 0.03	0.68 ± 0.04	0.7 ± 0.03	0.7 ± 0.06	0.68 ± 0.1	0.65 ± 0.09	0.67 ± 0.08	
	**D**	0.59 ± 0.11	1.1 ± 0.1	0.8 ± 0.09	0.7 ± 0.15	0.8 ± 0.07	0.9 ± 0.06	0.6 ± 0.08	0.9 ± 0.11	
	**p-value**	**0.06** ^*^	**0.001** ^*^	**0.055**	**0.9**	**0.005** ^*^	**0.26**	**0.02** ^*^	**0.06** ^*^	
**UREA (mg/dL)**	**A**	26 ± 0.7	35 ± 1.4	36 ± 0.8	23 ± 0.3	38 ± 1.5	23 ± 2	27 ± 1.0	23 ± 2.6	**20–45**
	**B**	23 ± 1.4	19 ± 0.7	24 ± 1.2	30 ± 0.9	38 ± 2.5	24 ± 3	33 ± 1.6	24 ± 3.2	
	**C**	25 ± 2	22 ± 3.4	25 ± 4.3	24 ± 4.1	27 ± 3.7	25 ± 2.9	27 ± 3.0	24 ± 3.6	
	**D**	24 ± 2.4	31 ± 3.1	36 ± 2.3	29 ± 1.9	32 ± 2.2	27 ± 3.4	35 ± 2.1	33 ± 2.7	
	**p-value**	**0.33**	**0.001** ^*^	**0.001** ^*^	**0.005** ^*^	**0.001** ^*^	**0.171**	**0.004** ^*^	**0.001** ^*^	

Two‑way RM‑ANOVA detected significant Group/Time effects at isolated time points (AST at 75 min, ALT at 45–60 min; *p* ≤ 0.05) with GG correction where appropriate. No patterns indicated clinically relevant hepatic or renal dysfunction under the tested single‑dose protocols.

## Discussion

Domestic rabbits exhibit elevated perioperative mortality rates (1.39%−4.8%) in contrast to dogs and cats (0.1%−0.2%), with cardiovascular and respiratory problems being the most frequently documented. Comprehending anesthetic risk variables is crucial to decrease mortality risks [17]. In this study we used three Pharmacological Context: Gabapentin (25 mg/kg, oral) as anticonvulsant with mild sedative and reflex suppression effects, enhancing anesthesia. In rabbits, it may reduce sympathetic tone, potentially lowering heart rate and blood pressure, with delayed onset (1.75 h) due to oral administration [10, 18]. Xylazine (5 mg/kg, IM) an α2-adrenergic agonist, causing sedation, analgesia, and muscle relaxation. In rabbits, it induces bradycardia, hypotension, and respiratory depression, with onset ~ 5–10 min and duration ~ 30–60 min [19], and Ketamine (35 mg/kg, IM), producing dissociative anesthesia with minimal respiratory depression. In rabbits, it causes tachycardia and maintains or increases blood pressure, with rapid onset (~ 2–5 min) and short duration (~ 20–40 min) [20].

Our analgesic data findings revealed the superiority of group (A) which provided the most sustained analgesia with a significant differences from groups (B), (C), and (D) at 15–90 min (*p* < 0.05), it may be due to gabapentin’s enhancement of ketamine’s analgesia, and supplemented by xylazine’s moderate analgesia, reflecting synergistic potency [21]. Group (D) provided the weakest analgesia with significant differences from group (A), (B), and (C) at most time points (*p* < 0.05), as ketamine alone provides transient analgesia in rabbits [22, 23].

Concerning the level of sedation; Group (A) showed the strongest, most sustained sedation, due to synergistic effects of xylazine which provides deep sedation [24], it may be due to dissociative sedation of ketamine, and gabapentin may enhances duration and depth. Significant differences from group (B) and group (D) at all time points, and from group (C) at 60–90 min (*p* < 0.001). Group (C) had a strong early sedation, matching group (A) initially, but waned faster this data agreed with [25, 26]. Xylazine drives sedation, with ketamine supporting, but lacks gabapentin’s prolonging effect.

Concerning the Assessment of anesthetic depth, which has been done by evaluating the response of animal reflexes and the degree of muscle relaxation; testing the reflexes response revealed the strong suppression of Group (A), achieving absent of all reflexes at 15–45 min, sustained minimal reflexes through 90 min. with sustained muscle relaxation, due to xylazine’s potent depression, ketamine’s rapid onset, and gabapentin’s prolongation [27, 28]. Group (B) revealed a moderate reflexes suppression and muscle relaxation, these data agreed with Van Elstraete et al. [29], who found that gabapentin enhances ketamine effect. Lacking the xylazine decreases the potency of the anesthetic regime. Group (C) showed strong early reflex suppression and muscle relaxation, then minimal reflexes response through 90 min. Xylazine/ketamine provided early potency but waned without gabapentin. Group (D) posed the weakest suppression and muscle relaxation, minimal reflexes at 15–60 min, returning to normal reflexes response by 75–90 min., These data agreed with [30] who stated that ketamine alone is insufficient for deep reflexes suppression and [31] who reported that ketamine alone is inadequate for effective muscle relaxation, posing a challenge in certain surgical procedures. Reflex endpoints and respiratory trends were contextualized using rabbit induction studies comparing ketamine–propofol ratios, which documented dose-related respiratory depression and validated our reflex methodology [32].

The anesthesia induction duration was observed to be the shortest in Group (A) driven by synergistic effects of gabapentin (pre-dosed, enhancing sedation), xylazine (rapid sedation), and ketamine (quick anesthesia). Group (B) had a moderate induction, slower than group (A) due to absence of xylazine. Gabapentin and ketamine combine for sedation, but lack xylazine’s potency [33]. Group (C) was like group (B), slightly slower. Xylazine and ketamine provide rapid sedation, but xylazine’s onset (~ 5–10 min) [19], so group (B) was slower than group (A)’s triple combination. Group (D) showed the slowest induction, this is agreed with [34] who stated that ketamine alone is less effective, requiring higher doses or time for full anesthesia.

*Cinar*, et al. [35], found that oral gabapentin (15 mg/kg) did not alter IOP/tear production/HPD but did modestly improve handling ease (muscle relaxation/partially closed eyes) in this study we use a higher dose of gabapentin (25 mg/kg) which can safely be used in rabbits with high plasma concentration 17.70 µg/ml after 1.75 h [36], this dose had good sedative effect which reflected on the recovery time. The longest recovery time was observed in group (A) reflecting prolonged effects of gabapentin (~ 4–6 h) [37] and xylazine (~ 30–60 min) [19, 24], compounded by ketamine. Group (B) had a moderate recovery, faster than group (A) due to absence of xylazine. Gabapentin extends recovery slightly beyond ketamine’s short duration. This data agreed with Jafarbeglou, et al. [38], who found that gabapentin’s anxiolytic properties appear to contribute to reduced induction time and prolong the recovery time when used before xylazine/ketamine anesthetic regime, while the data disagreed with Ferronatto et al. [39], who found that administration of gabapentin before anesthetic regime of (acepromazine/propofol) neither prolonged the recovery time nor decreasing the induction time in healthy cats. Although our results demonstrate reduced responsiveness to noxious stimuli, direct postoperative pain assessment using validated scoring tools in rabbits was not performed. Therefore, claims regarding surgical pain control should be interpreted cautiously.

Observing the vital parameters of the experimented rabbits which included (RR, HR, and rectal temperature) revealed significant differences between all tested groups. Group (A) which had a Profound muscle relaxation aligns with severe bradycardia, respiratory depression and hypothermia at 15–30 min, reflecting xylazine’s potent effects enhanced by gabapentin and ketamine. Group (B) with a moderate muscle relaxation corresponded to a milder vital sign change, indicating gabapentin-ketamine’s balanced profile. In group (C), a strong early muscle relaxation matched a moderate bradycardia, respiratory depression, and hypothermia, driven by xylazine. Group (D) which showed weak muscle relaxation aligned with tachycardia and stable respiration/temperature, reflecting ketamine’s limited sedative effect. Group (A&C) exhibited significantly lower respiratory rates at all sampling times than the other groups. This lower respiratory rate was attributed to xylazine’s pronounced respiratory depressant effects [40, 41]. Group (A) exhibited significantly lower heart rate values than other groups after anesthesia induction, the notably lower heart rate in Group (A) is in line with previous literature [22, 42–44] which associated with the dominant parasympathetic effects of xylazine on the cardiovascular system.

General anesthesia impairs thermoregulation in the central nervous system by obstructing vasoconstriction and lowering body temperature [11]. Research on general anesthesia in rabbits indicates a decline in body temperature, ascribed to compromised thermoregulation, less muscular activity, and lowered metabolic rates during anesthesia (Amarpal et al., [45]; Purohit et al., [46]). In accordance with existing data, comparisons among groups indicated that group (A & C) exhibited markedly lower body temperatures than the other groups.

Concerning the hematological result, all values remain within normal range in all groups. Group (A&C) showed reduction in Hgb and RBCs count (15–60 min) correlating to bradycardia and hypotension effect of xylazine, these data agreed with many previous studies [47, 48]. While Group (B&D) remain stable as gabapentin and ketamine have minimal effect on blood parameter [49].

AST is a broadly distributed enzyme present in a variety of tissues and organs, with the liver showing the highest activity. The rise in AST activity in serum is a sensitive indicator of liver injury [44, 50]. Group (D) has early AST elevations indicating that ketamine induced muscle stress [51]. Group (C) has spike at 75 min, suggesting delayed hepatic stress. However, all values are within normal in all tested groups. ALT has little tissue selectivity, making it ineffective for assessing hepatic damage in rabbits. For example, ALT levels in rabbit heart muscle and liver are identical, while rabbit liver has half the ALT levels found in dogs. ALT has a half-life of only 5 h in rabbits, compared to 45 to 60 h in dogs. Significant rises in ALT have been observed in circumstances of liver injury and necrosis, such as hepatic coccidiosis and hepatic lipidosis [52]. ALP is nonspecific enzyme in rabbits as it originates from many tissues, including bone, intestine, kidney, and placenta, as well as liver, with highest levels in intestine and kidney [52]. All ALP values of tested groups were within the normal range. Group (A&B) showed stable AST, ALT and ALP values which indicates the minimal effect of gabapentin on liver and heart.

Concerning CREAT and Urea, all values within normal range in all tested groups, Although the excretion of gabapentin was done through kidney, there was not any abnormal elevations in the values of CREAT and Urea in group (A&B) indicating the minimal effect of gabapentin on kidney when it is used at a single oral dose 25 mg/kg.

## Conclusion

In New Zealand White rabbits, oral gabapentin (25 mg/kg) given 2 h before induction and combined with xylazine (5 mg/kg IM) plus ketamine (35 mg/kg IM) was associated with faster induction, greater reflex suppression/muscle relaxation, and a longer recovery than ketamine alone or gabapentin-ketamine. The gabapentin-ketamine protocol showed less physiological depression than the xylazine-containing regimen, with intermediate effects on depth and duration. These findings should be interpreted considering the limitations of the scoring tools and the available monitoring.

## Data Availability

No datasets were generated or analysed during the current study.
